# Focus-Groups as Data Collection and Citizen Learning Simulation

**DOI:** 10.1177/14550725261503881

**Published:** 2026-11-06

**Authors:** Michael Egerer, Sébastien Berret

**Affiliations:** 1 Faculty of Social Sciences, 154260 Centre for Research on Addiction, Control, and Governance CEACG, University of Helsinki, Helsinki, Finland

**Keywords:** gambling, focus group, learning, policy, qualitative methodology

Large scale population level surveys are a common scientific tool to inform policy decisions, and addiction policies are no exception. Surveys are, however, bound to the notion that informants have answers ready to be collected as data. This might be less a problem regarding easy, clear-cut, and publicly present questions and topics, but becomes problematic for more complicated matters (Fishkin, 2006; Samuelsson et al., 2019). Inquiries on attitudes, opinions, or justifications of the regulation of addictive products and services might be such a matter. Surveys are also limited by researchers’ assumptions, which might lead to unforeseen variables or trends to fly under the radar. Qualitative methods help overcome these pitfalls by allowing a flexible research design through which researchers can account for phenomena they did not expect when formulating their research questions, while enabling participants to contribute actively to knowledge production (Knapik, 2006) and ensuring research is reciprocal rather than merely extractive (McKown & Stephan, 2025).

Focus groups are an interesting option in this regard. Focus groups are group discussions steered by a facilitator that often involve a collective activity (e.g., viewing a video clip or a commercial) and during which participants explore a given theme. Focus groups have been a popular method in health research (Wilkinson, 1998) and in qualitative research in general (Gehrmann et al., 2026). The distinguishing feature of focus groups lies in the explicit use of participants’ interaction to generate data: researchers try to encourage participants to engage with one another, often resulting in discussions going to unexpected directions and prompting the latter to reflect on their beliefs (Kitzinger, 1994). This method can also help people to clarify their thoughts when dissent appears in the group (Kitzinger, 1994). Focus groups go beyond the traditional roles between researchers and participants by prompting the latter to actively contribute to the process of analysis (Kitzinger, 1995). Thus, participants co-construct meaning (Wilkinson, 1998) because the group setting allows ideas to emerge through conversation. The process during which participants share their own views and compare them to those of others creates shared meanings and enriches the research by expanding the research topic (Morgan, 2012). Focus groups also allow participants to learn from these interactions, making this method generative and collaborative rather than extractive – and constitute therefore an ideal tool for research and democracy.

In the focus group study on the public image and function of the Finnish gambling monopoly (Egerer et al., 2018), we witnessed an interesting finding: The focus group discussions showed a considerably higher degree of criticism than the parallel population survey (Salonen et al., 2017). The focus group participants were recruited from these participants of the survey who indicated to be ready to be contacted for further studies. In the survey, the majority was satisfied with the gambling marketing of the state monopoly Veikkaus, whereas the participants of the focus groups considered the gambling advertisement as inappropriate, as encouraging people – especially minors and individuals experiencing gambling-related problems – to gamble. Furthermore, opinions concerning the availability of gambling, as well as options to regulate gambling, were in the focus groups more critical than what has been observed in the previous population surveys (Salonen & Raisamo, 2015). Retrospectively, the time of our interviews has been a turbulent moment in the public opinion on the regulation of gambling and gambling itself in Finland, with a rather uncritical public opinion on gambling (and indifference to its regulation) in the mid-2010s towards a more critical evaluation of gambling and the Finnish gambling monopoly, and particular its marketing (Grönroos et al., 2024). The critical appraisal of the monopoly found its culmination in 2021 with the book “Veikkauskratia” (a wordplay with the name of the Finnish monopoly “Veikkaus” and the Finnish version of the ancient Greek word “kratos” for power) written by a journalist, which questioned the monopoly's unfair practices in exploiting people by gambling in the name of the public good (Larsen, 2021). These developments were all in the making during the time that we conducted our interviews in 2017.

We argue that the focus group methodology might in fact be a valuable tool for the early identification of societal topics of importance and public opinion changes and thus a crucial complement to other study designs in informing (gambling) policy. The capacity for early identification lies in the characteristics of our focus group method, which combined interview stimuli, tasks, and questions, with a short info-leaflet about gambling and its regulation in Finland (Egerer et al., 2018) (Box 1).

Box 1. Information leaflet for the study participants (translated from Finnish).

**Background Information: Gambling in Finland**

− 80% of Finns have gambled at least once during the last 12 months
− 3.3% of population have gambling problems
− A gambler uses on average €11.30 per week for gambling
− Gambling generates approximately €1 billion per year for good causes (e.g., social organizations, culture, sports, veterans) [in comparison: e.g., alcohol taxes: approximately €1.36 billion per year]
− In Finland all gambling games are organized by the monopoly. Only Veikkaus gambling games are legal in Finland
− Many other countries have a so-called licence system. For example, in Denmark, there are 26 licences for Internet casinos, which private companies can apply for to offer Internet gambling legally in Denmark

References:

spillemyndigheden.dk/en/online-casinos

Suomalaisten rahapelaaminen 2015 (Terveyden ja hyvinvoinnin laitos)

valtionbudjetti.fi/

Veikkaus avustuskohteet (veikkaus.fi/yritys)

This leaflet sparked some discussion inside our research group. Are we putting something into our participants’ mouths, or are we enabling them to discuss the topic of gambling regulation credibly? In the end, based on our experiences in the pilot interviews, we decided to use this short form to provide some basic information, including the case of the Danish gambling regulation to offer an alternative to the often taken-for-granted status quo (i.e., the monopoly system). This information was intended to help the participants understand a complex matter. Compared to us researchers, we should not expect that the ordinary citizen would ponder on such issues night and day and would be aware about regulatory details and specificities. People rarely come with clear opinions and attitudes ready to the focus group interviews, but they shape them during the interview (Vaughn et al., 1996). This of course does not mean that a study's informant would be ignorant and would not know anything about the subject matters. It is also likely that persons dedicating their time to participate in a 90-min focus group have at least some interests in the topic, maybe also some knowledge. Many study participants showed indeed a surprisingly solid knowledge about the Finnish gambling regulation and were well versed in discussing such a complex matter in a group of peers. We experienced such skills also in other focus groups studies conducted by one of the authors (Egerer & Rundle, 2025), where the participants were in fact able to discuss complex topics like the brain disease model of addiction in a divert and competent manner. Qualitative research under a constructivist perspective expects focus group participants to come with their experiences and knowledge about the world into the focus group situation and then re-evaluate these in the light of the questions, tasks, and peers during the focus group (Sulkunen & Egerer, 2009). Witnessing this process is the collection of data in focus group. The process of referring to ones’ already existing knowledge to handle novel situations is elsewhere in fact simply called learning. Group learning has lately become the default learning method, at least in higher education (Biggs & Tang, 2011). The focus groups can be thus also seen as much as a learning event as they are a method for data collection. This type of data collection might be an artificial situation but nevertheless brings not-now and not-here situations into the presence of the focus group enabling natural interaction and learning (Demant, 2012). One could thus say that our focus groups similarly simulated the citizen learning process or the public debate in gambling regulation in miniscule.

There were three elements that underline the overlap of data collection and learning in our focus group study. One explanation for the higher level of criticism was offered on several occasions by the participants themselves: they actively reflected on their learning process regarding their discussions concerning the presented commercial of the Finnish gambling monopoly (Box 2).

Box 2. Example quotation on active reflection.

*Male participant 1: Yes, one thing to remember is that you are not analysing these commercials when watching them at home*

*Female participant 1: Hardly*

*Male participant 1: That something sticks to your sub-consciousness, which of course might influence your behaviour later and without knowing what did it [the behaviour change] cause. But isn’t that the whole idea [of marketing]*

*Female participant 1: Sticks somehow to your mind*

*(Group 9, occasional gamblers)*

Second, there were also instances of peer learning, such that participants reacted in their opinions on input from other participants (Box 3).

Box 3. Example quotation on peer learning.

*Male participant 1: Just now I realise, as we spoke about how gambling is organized abroad, that it is very liberal in Finland, that here there are [gambling machines] in shops, really everywhere; that abroad there are these small sleezy looking casinos, where you need to go if you want to gamble, the threshold is clearly higher.*

*Female participant 1: Yes, and they [the machines] are not necessarily everywhere in the shop, but you need to travel a bit. I used to live in France for one or two years and there you can maybe play the lottery or some scratchcards in a kiosk kind of place, but if you want to gamble at a gambling machine you need to go to a casino. And in their understanding, there is something magic [special] about visiting a casino. (…) (Group 2, occasional gamblers)*

Finally, there were also instances when participants made direct use of the information leaflet thus, they augmented their knowledge with the novel information available during the focus group.

As the example quotation (Box 4) shows, the leaflet tended to sharpen participants’ arguments rather than change their opinions, mitigating our initial concern that the leaflet would steer the discussions into a certain direction. At least, it did not seem to be the reason for a more critical stance on the Finnish regulation of gambling at the time.

Box 4. Example quotation on use of information leaflet.

*Male participant 1: Yes, absolutely, that somehow it justifies it [the monopoly], that if you let private businesses do it, yes, I would not even trust it*

*Male participant 2: I am just thinking that how these licences needed to be that it would be the same as with Veikkaus [Finnish gambling monopoly]. That they would need to put similar amounts towards good causes, or how these licences would be, that in Denmark obviously, they grant these licences that how it works there, but…*

*Female participant 1: But a monopoly is good in monitoring; and then also where the money goes, it is for the common good and it is monitored and acceptable*

*(Group 4, experienced gamblers)*

The use of focus group interviews as a form of data collection and as citizen learning simulation might be limited by the specific context of our study and might not work in the same way in other countries or contexts. The focus group setting can also affect the answers obtained, as participants are likely to express more extreme opinions during the data collection than in real life (Boulton & Kitzinger, 1994). Then again, this limitation of the focus group methodology might explain why it is more effective at identifying emerging trends at an early stage. Focus groups are known to excite more emotional engagement and become more explorative (Kitzinger, 1995). The focus group situation can thus bring forward something not yet fully articulated but bubbling under inside a society. Obviously, this also asks for caution with the focus group data in order avoid overrating particularly strong assertions. Recognising that focus groups are not only a form of data collection, but also learning events raises furthermore an ethical question: what do the participants take home as their new understanding of the topic (in our case the regulation of gambling in Finland) and what is our (i.e., the researcher's) role in such learning? Consideration given to the impact of methodological approaches on the participants is common in research (McCambridge et al., 2014). In the context of a focus group study, this matter demands from us researchers accuracy in the information provided to the participants, and a balance between detail and easy understandability that must be evaluated before the research is conducted. In some cases, it might also make it necessary to integrate a debriefing phase at the end of the focus group to correct outright wrong or dangerous claims made during the focus group discussion (cf. Hyde et al., 2005). While we could not observe such claims in our focus group studies, a claim such as it would be legal to open one's own casino, would need to be clarified as wrong if not done so by the other focus group participants.

We are not claiming the answers and data acquired by the focus groups being ‘better’ or more genuine than the ones obtained in population surveys – such a statement would stand in direct contradiction towards the bases of a reflexive qualitative research philosophy (Spencer et al., 2014). The results in our focus group study (Egerer et al., 2018) offered an important contribution towards the debate on attitudes towards gambling and gambling regulation in Finland. While the survey most likely caught the overall opinion landscape at the time, the focus groups rather identified early developments and opinion forming. A policy informed solely by population surveys risks drawing significantly different conclusion than a policy being balanced by the results from a focus group study. What might be elsewhere frowned-upon as biased data might in fact offer additional credibility (Guest et al., 2012) by enquiring on informed opinions, when compared to the data from survey data collection methods. The latter methods often leave the less informed participants, or participants without a formed opinion, to giving ad hoc answers with low informative value.

Finally, understanding focus group studies as simulated citizen learning and using the results in informing policy are pivotal. It is also ethical by facilitating democratic decision making because it has the potential to increase citizen participation in the process in a real informed sense, instead of asking purely pre-defined “top-down” questions (Burgess, 2014; Fischer, 1993).
